# Integrated Nanophotonic Waveguide-Based Devices for IR and Raman Gas Spectroscopy

**DOI:** 10.3390/s21217224

**Published:** 2021-10-30

**Authors:** Sebastián Alberti, Anurup Datta, Jana Jágerská

**Affiliations:** Department of Physics and Technology, UiT the Arctic University of Norway, 9019 Tromsø, Norway; anurup.datta@uit.no (A.D.); jana.jagerska@uit.no (J.J.)

**Keywords:** integrated sensors, waveguides, absorption spectroscopy, Raman spectroscopy, gas sensing

## Abstract

On-chip devices for absorption spectroscopy and Raman spectroscopy have been developing rapidly in the last few years, triggered by the growing availability of compact and affordable tunable lasers, detectors, and on-chip spectrometers. Material processing that is compatible with mass production has been proven to be capable of long low-loss waveguides of sophisticated designs, which are indispensable for high-light–analyte interactions. Sensitivity and selectivity have been further improved by the development of sorbent cladding. In this review, we discuss the latest advances and challenges in the field of waveguide-enhanced Raman spectroscopy (WERS) and waveguide infrared absorption spectroscopy (WIRAS). The development of integrated light sources and detectors toward miniaturization will be presented, together with the recent advances on waveguides and cladding to improve sensitivity. The latest reports on gas-sensing applications and main configurations for WERS and WIRAS will be described, and the most relevant figures of merit and limitations of different sensor realizations summarized.

## 1. Introduction

Both IR absorption spectroscopy and Raman scattering spectroscopy are nowadays routine characterization techniques that are available in most organic and material processing labs, as well as in the industry. These techniques provide specific information about molecules or chemical functional groups in a fast, non-invasive, and reliable manner and have been used to identify compounds, follow reactions, and track absorption processes. Their wide variety of applications include environmental monitoring, (i.e., not only the monitoring of pollutants and greenhouse gases but also real-time monitoring of anesthetics and respiratory gases during surgery), explosives detection, medical diagnostics, and even the authentication of paintings, aside from their widespread use in research and industry [1,2,3,4].

Absorption and Raman spectroscopy, although providing similar information, are complementary techniques as the rotational-vibrational signal, silent in Raman scattering, can be highly noticeable in absorption experiments, and vice versa. It is therefore of no surprise that both techniques have been developed simultaneously for similar purposes. They exhibit different configurations according to the nature of the sample, i.e., for liquids, thin films, powders, or gases. Gas detection based on Raman and absorption spectroscopy relies heavily on the boost in sensitivity through the use of resonant cavities and multipass cells that increase the path length and, hence, the interactions of the beam with the analyte. Such cells and cavities have, almost exclusively, been realized by free-space beams and bulk optics; as a consequence, the standard high-end spectroscopy instruments still remain bulky, and samples often need to be collected and analyzed in the laboratory.

Recently, portable tunable laser absorption spectroscopy (TLAS) instruments for trace gas analysis have been developed by Aerodyne Research Inc., LI-COR, Picarro, IRsweep, and others. These, being typically packaged as 19-inch rack modules or of the size of a large suitcase, have been used for in situ monitoring in mobile vehicles, including airborne field campaigns. Shoebox-sized and only 3 kg in weight, instruments offered by Aeris Technologies followed, as well as a compact, 2.1 kg and battery-powered sensor developed by Empa for measurements aboard unmanned aerial vehicles (UAVs) [5]. Alternative portable devices include quartz-enhanced photoacoustic spectroscopy (QEPAS) [6], a variant of photoacoustic spectroscopy (PAS) in which the microphone is replaced by a quartz tuning fork. The instrumentation of Raman spectroscopy includes handheld Raman spectrometers, produced by Bruker, Thermo Fisher Scientific, and other companies.

Integrated on-chip devices appear to be the next logical steps to decreasing the size even further while keeping the advantages of molecular selectivity and sensitivity as offered by IR and Raman spectroscopy techniques. This will ultimately require the monolithic integration of a laser and a detector, with a photonic chip replacing a classical gas cell. In most cases, this photonic chip is constituted by a high-finesse photonic cavity, or a long, single-mode waveguide often curled into a spiral and tightly patterned on a photonic chip. Both these configurations enable long optical pathlengths for high sensitivity while keeping a minimal footprint, e.g., a chip size of the order of one square centimeter. In such waveguide-based sensors, the guided light penetrates the evanescent field outside the waveguide core and probes a sample close to the waveguide surface. Molecules present within the evanescent field will absorb light or generate Raman scattered light that will couple back into the waveguide modes. The respective interaction pathways give rise to analytical techniques that are also known as waveguide infrared absorption spectroscopy (WIRAS) and waveguide-enhanced Raman spectroscopy (WERS).

In this review, we will discuss the major advances made in waveguide-based absorption and Raman spectroscopy for gas detection. The first section will address the main components developed so far to achieve the miniaturization of integrated sensing devices: light sources, waveguides, cladding, and detectors. In particular, the materials and designs that are proposed to decrease losses while increasing light interaction with the surrounding environment will be described. Additionally, a brief description of cladding as a strategy to improve sensitivity will be provided. The second section will focus on the integrated Raman and IR absorption sensors reported so far. The main sensor configurations will be introduced, and the latest applications will be discussed, discriminating between air-clad and functionalized/clad waveguides. Finally, a technology map will compare the performance of individual WIRAS and WERS sensors reported to date.

## 2. Efforts toward Miniaturization

### 2.1. Light Sources

#### 2.1.1. IR Absorption Spectroscopy

One of the most widely used IR absorption spectroscopic techniques is based on tunable laser absorption spectroscopy (TLAS), which relies on a narrow-band light source such as a single-mode laser, where the wavelength can be carefully tuned to overlap with an absorption peak of the target analyte. This strategy has been applied most particularly in high-end trace gas sensors in the traditional gas cell configurations and their associated derivations involving optical cavities (such as cavity ringdown spectroscopy, cavity-enhanced absorption spectroscopy, and noise-immune cavity-enhanced optical-heterodyne molecular spectroscopy). Advances in MIR photonics over the last two decades have brought about high-quality laser diode sources based on interband cascade lasers (ICLs) [7,8], quantum cascade lasers (QCLs) [9,10], vertical-external-cavity surface-emitting lasers (VECSEL) [11,12,13], and frequency comb lasers [14,15]. The possibility of integrating light sources into chip devices has made them particularly suited for use in waveguide-based spectroscopy devices [11,16,17]. Excellent stability, tunability and the narrow line-width of these lasers have enabled IR laser absorption spectroscopy of unprecedented sensitivity and specificity, making TLAS gas sensors a powerful alternative to conventional FTIR and NDIR spectroscopy. While IR laser absorption spectroscopy has traditionally focused on single-species detection, extending the tunable wavelength range enables multispecies detection with a single laser source. Dual-wavelength distributed feedback (DFB) QCLs or lasers implementing Vernier-effect tuning are integrated light sources that have turned this into a reality, thus expanding the range and applicability of such lasers [18,19,20]. Further extension of the tunable range of QCLs has used a Fabry–Perot QCL chip in an external-cavity (EC) system, where the laser could be tuned across the whole gain curve. In addition, DFB QCL arrays have been used to extend the tuning range, making it possible to electrically switch between emission frequencies [21,22,23].

On the other hand, broadband-coherent sources, such as supercontinuum lasers [24,25], have also been used for MIR spectroscopy. The development of the waveguide-based generation of a supercontinuum [26,27,28,29] has provided the perfect impetus for the realization of compact on-chip light sources. The broadband nature of these supercontinuum sources allows the simultaneous probing of several analytes but this also translates to low selectivity; hence, complex post-processing algorithms are needed to demarcate between the overlapping absorption spectra across different analytes [30,31]. Often, configurations in conjunction with wavelength filters or spectrometers are needed in order to maintain selectivity. While these laser sources have good coherence and adequate power output, making them well-suited for detecting trace quantities of gas, they are generally very complex to fabricate and are hence quite expensive as well.

Some state-of-the-art light sources include nanolasers based on plasmonic structures [32] and metamaterials [33], which hold a lot of promise regarding the realization of compact light sources. Many of these metamaterial-based light sources offer an easy way to control the wavelength of the emission through scaling the unit cell design to longer or shorter wavelengths, as compared to other light sources [34,35]. In contrast to this, incoherent light sources, such as MIR light-emitting diodes (LEDs), have also received attention due to their small size and low power consumption [36,37,38]. Among the MIR LEDs, super-luminescent light-emitting diodes (sLEDs) offer a unique combination of high brightness, good beam directionality and broadband capability [39]. However, they have been limited to wavelengths smaller than 5 μm due to the poor efficiency of light emission at longer wavelengths [40]. In particular, on-chip LEDs on SOI that is fabricated through the heterogeneous integration of InP membranes help to couple the light efficiently to a single-mode waveguide and help to avoid high coupling losses and high packaging costs [41]. Another approach is the direct material integration of active emitters, such as quantum dots, within the waveguide itself. As demonstrated in a silicon nitride platform with embedded quantum dots, this represents an elegant solution for generating waveguide source light with high-mode coupling [42].

Thermal emitters have emerged as one of the latest and most promising means of generating MIR radiation. In this case, plasmonics and metamaterials principles are used to design nanostructures with high spectrally selective absorptivity. Kirchoff’s law then requires the radiation from these structures to emit in the same spectrally selective region. Certain MEMS-based structures and micro hot plates have been demonstrated as offering a good light, suitable for analyte sensing, due to their energy efficiency, fast modulation capability, and CMOS-compatible processing steps [43,44,45].

#### 2.1.2. Raman Spectroscopy

Excitation light sources for Raman spectroscopic systems are typically high-power monochromatic laser sources, operating with more than 50 mW output power in the visible or in the near-infrared, typically at 785 nm or 1064 nm wavelengths. While external laser sources have primarily been used for Raman spectroscopy, recent advances in silicon photonics have made possible a wide variety of compact and chip-based lasers [46], which would ultimately pave the way for an integrated Raman system. However, to date, there are only a few examples of miniaturized laser sources combined with on-chip Raman systems. The difficulties in limiting the realizations of such systems arise from the requirement of a high-power monochromatic light source, and the difficulty in separating the pump and the scattered Raman signal with a high-enough extinction ratio on a chip. The strict requirement of high laser power is motivated by the inherent weak scattering efficiency of the Raman signal [47]. Several strategies have been proposed to increase the strength of the Raman signal, such as confining the light to a small volume, particularly through the use of nanophotonic platforms, such as enhanced hotspot formation through the employment of metallic nanostructures in surface-enhanced Raman configuration [48,49] or through the use of slot waveguide platforms [50,51]. Working in a lower wavelength regime also helps to increase the scattered signal intensity since the scattering varies inversely as the fourth power of wavelength. However, this is often accompanied by the presence of unwanted fluorescence background [52]. 

Large-throughput spectrometers have traditionally been a major and integral part of a Raman spectrometer setup (see Section 2.4). A recent work by Atabaki et al. has demonstrated the concept of using tunable lasers as a way of eliminating the spectrometer from a Raman setup, through a method called swept-source Raman spectroscopy (SRSS) [53]. In this case, an excitation laser with only a few mWs of power was tuned in combination with a narrow bandpass filter on the detector side, which resulted in significantly high optical throughput compared to benchtop and compact handheld dispersive Raman spectrometers. A MEMS tunable laser was used, based on the concept of vertical-cavity surface-emitting lasers (VCSELs), thus demonstrating the suitability of VCSEL lasers for use in miniature Raman sources, as was also proposed earlier [54,55]. This work represents a major step toward the realization of miniature light sources for Raman spectroscopy.

### 2.2. Waveguides

Waveguides for sensing, including those for WERS and WIRS, have been evolving to guarantee minimum losses and high light–analyte interaction. Losses can be divided into the absorption of the material, leakage to the substrate, bending losses, and scattering losses due to fabrication or material imperfections (inhomogeneity or crystal grains). To minimize them, a proper choice of both the material and the waveguide design is crucial. 

Aside from the transparency of the waveguide material in the targeted wavelength range, refractive index, photo-stability up to high intensities, low level of fluorescence or Raman background, toxicity, availability/cost, and ease of production are the main selection criteria. From the great variety of materials proposed, materials compatible with CMOS/mass production, such as silicon, germanium, silicon oxide and silicon nitride, are the most common [56]. Nevertheless, a wide range of other materials has been reported, including polymers (i.e., photoresists and Teflon) [57], halides, chalcogenides (i.e., CaF2, NaBr and ZnSe) [28,58], oxides (i.e., alumina, titania and tantala) [59,60], diamond (due to its advantages for quantum photonics) [61,62], or InGaAs [63]. Recently, a review on waveguide materials has been published by Yadav and Agarwal [64].

Doped silica (UV-written) and silicon nitride over a silica bottom cladding have been the main materials used for on-chip waveguiding in the visible- to the NIR range; however, alternatives such as tantalum pentoxide have recently emerged. These materials have also been used, to a lesser extent, in the MIR, despite the presence of residual O-H and N-H groups that limit their transparency in certain frequency bands. For MIR applications, silicon on silica (i.e., silicon-on-insulator, SOI) and, less frequently due to increased costs, germanium on silica (germanium-on-insulator, GOI), have been the materials of choice due to their transparency at longer wavelengths. SiGe alloys possess the highest refractive indices of all the CMOS-compatible materials mentioned above and can, in addition, be doped in order to tailor their refractive index. The high refractive index has also been shown to be highly useful in avoiding mode leakage into the substrate, enhancing the electric field at the waveguide interface and, thus, the light–analyte interaction. Besides SOI and GOI, silicon on nitride, silicon on alumina, and germanium on silicon (or silicon-germanium alloy on silicon) have been proposed as novel waveguide alternatives in MIR, due to their capability of avoiding absorption by silica bottom cladding, especially above 3.5 μm [65,66]. Diamond also appeared recently on the scene as an ideal material with a transparency range from 0.22–20 μm; nevertheless, its applications are limited due to the difficulty of processing and high cost [67].

The processing of these materials into photonic waveguides has also developed greatly during the last decades, not only to account for more complex designs and profiles but additionally to decrease losses. While the homogeneity of the materials is highly dependent on the deposition technique and post-treatment, the surface and mainly the sidewall roughness are subject to the etching protocol that is followed. The former will decrease bulk scattering, the latter, the surface scattering. A great number of deposition and etching protocols are already available in the literature and depend greatly on the materials, the etch rate, selectivity and profiles, or the design. We will not go further into the topic, but we do encourage our readers to find further information in the work of William et al. [68].

Finally, the waveguides’ design is crucially important when high sensitivity to the surrounding environment is targeted. Unlike waveguides developed for communication purposes, waveguides for gas sensing need to ensure high light–analyte interaction, assuming the strong presence of the optical field outside the solid waveguide core. The amount of this interaction can be described using the evanescent field confinement factor, Γ, which is defined as in [69]:

(1) Γ=(ngRe{ncl})∬cl ε|E|2dxdy∬−∞∞ε|E|2dxdy

The absorption along the waveguide length is then given by a modified Lambert-Beer law:

(2) $$I=I_{0}\exp\left[-\alpha \Gamma_{\;}L\right]$$

Here, n_g_ is the group index, *n_cl_* is the cladding’s refractive index (equal to approx. 1 in the air), ε(*x*, *y*) is the permittivity, *E*(*x*, *y*) is the electric field, α is the bulk absorption, and *L* is the length of the waveguide. It is important to stress that the absorption not only depends on the evanescent field fraction but also on the waveguide dispersion through the group index n_g_. Reporting only the evanescent field fraction and omitting the effect of dispersion is a common misconception in the literature, making it difficult to quantify and compare the light–analyte interaction across the different waveguide platforms reported in the literature.

Besides the confinement factor Γ, the sensitivity of the waveguide is also determined by the physical path-length of the waveguide L that is typically limited by the waveguide loss. Therefore, the ratio between the evanescent field confinement factor and the propagation loss was introduced by Kita et al. [70] as an additional figure of merit that fully determines the sensing performance of the waveguide. Both the confinement factor and the losses will be dependent on the material and the processing, as well as on the waveguide design [71].

**Figure 1:**
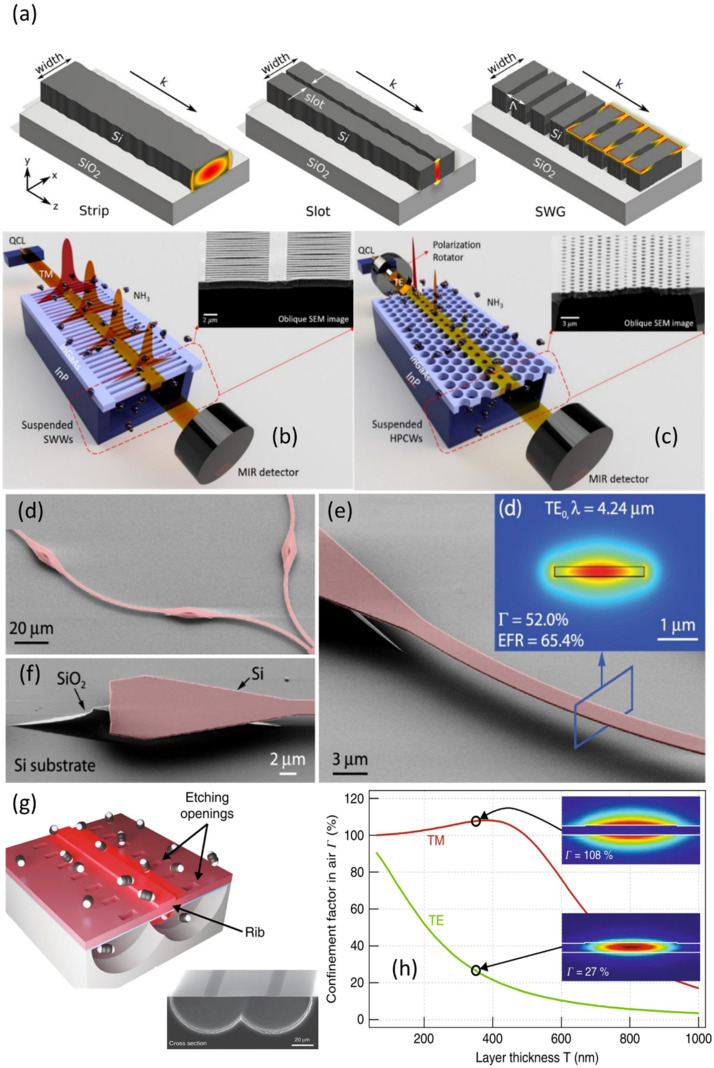
Different waveguide designs reported for WIRAS: (**a**) Strip, slot and subwavelength grating waveguides (SWG) (reproduced with permission from reference [70] © 2021 Optical Society of America). (**b**) Suspended subwavelength grating waveguide and (**c**) suspended photonic crystal waveguide. The insets show SEM images of the fabricated structures (reprinted with permission from reference [63], copyright 2020 American Chemical Society). (**d**–**f**) Suspended waveguides on pedestals; the inset in image (**e**) shows the cross-section and the electrical field distribution (reproduced with permission from reference [73], (© 2021 Optical Society of America). (**g**) Schematic and SEM image of a self-standing rib waveguide. (**h**) Simulated confinement factor of the waveguide in (**g**) at TM and TE polarizations, as a function of layer thickness, while the inset shows the electric field distribution at a thickness of 350 nm. Reproduced from reference [69], licensed under a Creative Commons Attribution 4.0 International License http://creativecommons.org/licenses/by/4.0/ assessed on 9 September 2021.

The most common waveguides reported for sensing can be classified into five different designs: rib, strip, slot waveguides, sub-wavelength gratings and photonic waveguides [72]. Rib and strip waveguides can be realized swiftly in one step with UV lithography and easy-etching protocols. The former is characterized by a shallow step defining the waveguide, with a small side-wall area and, therefore, little surface scattering compared to other designs. The strip waveguide (Figure 1a) is etched all the way down to the bottom cladding and exhibits more scattering loss but, unlike rib waveguides, it allows scientists to confine light tightly in the horizontal axis, resulting in minimal bending loss. According to Kita et al., who compared the performance of strip, rib, and slot waveguides for sensing, the strip waveguide is the preferred geometry for bulk absorption sensing and refractometry and is comparable in performance to other, more complicated, geometries for surface-sensitive refractometry and absorption sensing [70].

Slot, subwavelength grating (SWG), and photonic crystal waveguides have been reported as alternative designs that are capable of increasing the interaction with the surroundings by several times. Slot waveguides consist of two strips of high-refractive-index materials, separated by a subwavelength-scale low-refractive-index slot region that strongly confines light (Figure 1a). This design presents a light–analyte interaction more than 5 times larger than strip waveguides, which is highly desirable for gas sensing, while material losses are reduced due to the low intensity of the electric field in the material [70,74,75]. This design presents a good compromise between simplicity, air confinement factor, losses, and costs, and has been tested experimentally many times for both IR and Raman spectroscopy [76,77]. Despite the advantages, this design requires electron beam lithography in most cases to pattern the slot, which is typically of the order of 100 nanometers, and additional care needs to be taken during etching to guarantee a well-defined slot and little roughness [78]. 

A SWG waveguide (Figure 1a,b) is based on a periodic arrangement of two different materials having a period that is much smaller than the wavelength of light. It is characterized by field distribution with an air confinement factor 4–5 times higher compared to the strip waveguide [70]. Although, theoretically, no losses are expected from the design, the experimental propagation loss is normally above 2 dB/cm, due to imperfections arising during fabrication, particularly surface roughness and variability in the size of the waveguide segments [79].

Photonic crystal waveguides are distinguished by their ability to slow down light, i.e., to reduce the group velocity of the propagating waveguide mode as a result of coherent scattering on the photonic crystal lattice. Group velocity reduction by factors of between 1.5 and approx. 100 have been reported, and a corresponding increase in the interaction with the analyte has been observed in sensing experiments, both at NIR [80] and MIR wavelengths [81]. These waveguides are commonly formed by a linear defect in a photonic crystal lattice, patterned into a high-index dielectric membrane (see Figure 1c). The main drawback can be attributed to the difficulty of fabrication, a sensitivity to disorder that may lead to spectrally uneven enhancement, and an increased surface area that brings greater surface scattering and reflections [82]. Using slow light increases analyte-field interaction but, at the same time, it increases the interaction with the material, including absorption and scattering. In most demonstrations, the waveguide lengths are thus limited to hundreds of micrometers or, at most, millimeters.

To improve the light–analyte interaction even further, the bottom cladding can be removed either partially or completely. Partial bottom cladding removal was used to realize air-suspended waveguides supported by pedestals [49,83] or pillars [73,84] (Figure 1d–f). Complete cladding removal results in self-standing rib waveguides (Figure 1g), sub-wavelength grating waveguides (Figure 1b) [85], and photonic crystal waveguides (Figure 1c) [63]. Air-suspended structures appeared recently in the literature, exhibiting the largest reported confinement factors surpassing 100% (Figure 1h) [69] and reduced propagation losses [69,73]. By etching away the material beneath the waveguide, absorption due to the bottom cladding can be completely removed, leakage to the substrate avoided, and the volumetric interactions with the surrounding analyte increased [86]. Additionally, the lack of bottom cladding is well-suited for TM polarization which, in thin suspended waveguide designs, has minimal electric field overlap with the core material. This further increases the evanescent field confinement and decreases losses attributed to absorption in the constituent materials. Despite these advantages, the waveguide processing is complex and requires several lithography and etching steps; the structures are rather fragile, necessitating careful handling; and monolithic integration with laser sources and detectors appears more challenging than for waveguides supported by solid bottom cladding.

In order to achieve the lowest possible detection limits, the increase in sensitivity has to go hand-in-hand with the reduction of noise. An important noise source in integrated photonic circuits is a so-called interferometric noise, arising due to reflections from facets or defects, manifesting itself as spectral fringing in transmission. Such noise may interfere significantly with the recorded spectrum. For this purpose, antireflection coatings on the waveguide facet [87], the use of subwavelength gratings on the waveguide facet [88], or the use of appropriate signal processing algorithms [89] have been proposed to reduce the effect of the fringes. Substantial fringe reduction has also been achieved in air-suspended waveguides, characterized by strongly delocalized guided modes with an effective mode index close to unity. This automatically minimizes reflections at the facets or structural defects of the waveguide, leading to clear spectral transmission that is free from interferometric noise [69]. 

Another important point to consider in the context of high-index contrast waveguides for IR absorption spectroscopy is the possibility of saturation of the absorption signal, due to the intrinsically high intensity of the strongly confined guided modes. This typically occurs at high laser powers, in combination with intense absorption lines, where the excitation rate of the molecules can become faster than their relaxation rate [90]. To date, the literature mentions only sporadically the effects of saturation in waveguides, and detailed theoretical description is entirely absent. However, a waveguide design with a strongly delocalized field would mitigate this effect.

### 2.3. Cladding

Functional coatings, ranging from monolayers to films of several micrometers thick, have been used for decades as a route to increasing the sensitivity and/or specificity of integrated (bio)sensors. These layers work as molecular recognition coatings, serving as a solid-phase enrichment matrix for the targeted analyte, while simultaneously excluding undesirable molecules and avoiding unspecific binding. In other words, these layers enhance the signal to noise ratio by decreasing the background signal due to unspecific binding, they reduce the cross-interference with other molecules, and, at the same time, they increase the concentration of the targeted analyte relative to that of the surrounding media (i.e., solutions, atmosphere). The design of the coating layer can be adapted for a specific molecule through recognition sites, partitioning, and charge- or size exclusion [91]. This strategy has become of the utmost importance for highly sensitive transducers with low selectivity or specificity and has been adopted in a number of chemical-sensing devices such as opto-chemical, electro-chemical, plasmonic sensors, and refractive index waveguide-based sensors [92,93,94,95,96]. In the latter case, the analyte either increases the refractive index of the cladding or motivates a change in the thickness of the cladding itself [97,98,99], which is then detected by a phase-sensitive device such as a ring resonator, Mach-Zehnder interferometer, or a Bragg grating [95,97,100]. The advantages of functional cladding have also proven to be highly useful for already selective transducers, such as Raman and IR spectroscopy sensors, as a means to increase the sensitivity by analyte up-concentration and the reduction of the background signal in complex matrices.

The enrichment cladding layers can be oxides, polymers, silanes, specific biological molecules, or composite materials where more than one element is present. Polymers have been widely used due to simple processing, availability, tuneability (functional groups, molecular weight, ramification, backbone structure, or crosslinking degree), and their behaviour as extraction materials, with their enrichment properties mainly dependent on their polarity, free volume, pore size, and pore distribution [101]. A great variety of polymeric materials have already been investigated, including polyisobutylene [102], ethylene/propylene copolymer [103], low-density polyethylene [104], Teflon^®^AF [105], poly(dimethylsiloxane) [106,107], poly(acrylonitrile-co-butadiene) [107], poly(styrene-co-butadiene) [107], poly(vinyl chloride) [108], polystyrene, and poly(methyl methacrylate) [83,109,110]. Among these, fluorinated polymers have shown good transparency up to MIR wavelengths due to the substitution of C-H bonds, high free volume, and outstanding thermal and mechanical properties [57]. Although mostly used on waveguide-based refractive index sensors and ATR crystals, some specific polymers have been tested on integrated single-mode waveguides for Raman and IR absorption spectroscopy (see Section 3.2.2 and Section 4.2.2). 

Mesoporous inorganic and mesoporous hybrid inorganic-organic cladding, based on sol-gel chemistry, represent a robust alternative to polymers [111]. These materials are equally capable of providing partitioning for custom analytical tasks, while they exhibit advantageous optical, dielectric and thermal properties [112,113,114,115]. Mesoporous inorganic-based materials show robust mechanical and chemical stability capable of sustaining harsh environmental conditions such as high-energy radiation, acid, or alkaline media, as well as oxidative chemicals. Furthermore, these materials have tunable pore volume that can surpass 50% and a decreased response time (in the order of seconds) in comparison to many polymers (typically in the order of minutes). Optical losses in VIS-NIR in these materials are generally low, due to their amorphous structure and small pore size [111]. Nevertheless, the transparency at longer wavelengths suffers due to OH groups and the adsorption of water on the large surface area of the pore network. In addition, clad sensors normally need calibration due to variations in the material properties attributed to minimal changes in the process/environment.

Although cladding brings about improvements in specificity and sensitivity, calibration is mandatory for clad systems as the up-concentration factors are difficult to quantify analytically. Cladding properties may also change over time, due to phenomena such as thermal instability, dehydration, or reconfiguration. Increased response time and aging are among other limitations of clad sensor systems, as well as the potentially reduced reversibility of the system after exposure to the analyte, discriminating between disposable and reusable devices.

### 2.4. Detectors: Single Pixel, Arrays, Spectrometers

IR absorption spectroscopic systems primarily use single-pixel detectors in combination with monochromatic tunable laser sources. Here, the spectral selectivity comes from scanning over an absorption feature with a narrow linewidth of the laser rather than from a spectrally sensitive detection unit.

Among chip-integrated single-pixel detectors, group IV materials like silicon or germanium or a combination of III–V materials, such as InGaAs, InGaAsSb, InAsSb, PbTe, GaSb, and InP have been used as platforms for NIR and MIR sensing [64,65]. While Si can only absorb up to about 1.1 μm, ion implantation or introducing lattice defects or external agents can improve the detection range of Si-based photodetectors up to 2.4 μm [64]. The entire MIR spectral range can be covered by mercury cadmium telurite (MCT) detectors, which are also most widely used for MIR sensing in bulk spectrometers. However, due to a strong lattice mismatch, they are unsuitable for integration in chip-based systems. Besides this, 2D material-based detectors, such as black phosphorous, for up to about 4 μm [116,117,118,119] and semi-metals, like graphene, for a longer wavelength [120] have also been used as photodetectors. Lead chalcogenides, in particular PbTe, have also received significant attention for MIR photodetection due to their easy deposition process, excellent stability, and low cost. However, the above-listed photocarrier generation-based techniques for IR light detection inevitably suffer from high dark current, particularly when they are biased and, thus, they require significant cooling to achieve high sensitivity. This issue has been partially addressed by detectors with an engineered band structure such as superlattice detectors, quantum cascade detectors (QCDs), and interband cascade detectors (ICDs), which have received much attention due to their high detection sensitivity at room temperature [121,122,123].

The recent trend in the integrated detector domain has moved toward MEMS-based IR detectors, such as thermopiles, microbolometers, and pyroelectrics, which can work even at room temperatures and have detection capabilities across a longer wavelength range [124,125,126]. However, they still lag behind photodetectors in terms of sensitivity and have slower response times.

For broadband sources, which have been used for both IR spectroscopy and Raman spectroscopic systems, spectral discrimination is done at the detection side through one of the following systems: (i) miniaturized dispersive optics; (ii) narrowband filters; (iii) Fourier transform-based detection devices; and (iv) reconstructive spectrometers [127].

Dispersive optics has been the most widely used detection scheme, where dispersive optical elements, such as gratings, slits or arrays, spectrally separate the incoming broadband light and send it to either a single pixel detector or a detector array. While the usage of a single pixel detector is low cost, the setup requires the capability to scan across the spectrum, making the process slow and often prone to complex alignment requirements. The usage of an array of detectors makes the scan significantly faster but at a higher cost. The majority of dispersive spectrometers operate in the visible wavelength range, which makes them suitable for use in a Raman spectroscopic system. Infrared spectroscopy, on the other hand, most often relies on the principle of Fourier transform spectroscopy, its advantages being the ability to use only one detector, the simultaneous collection of spectral information (also known as Fellgett’s advantage), and higher optical throughput due to the elimination of optical slits. Compact Fourier transform spectrometers have been realized through miniaturized Michelson or Fabry–Perot interferometers [128,129,130], micro-electromechanical systems (MEMS), or micro-opto-electromechanical systems (MOEMS) [131], MEMS-based digital micromirror devices (DMD) or linear variable filter arrays [132]. For photonic integrated sensors, spectral discrimination can be implemented using waveguide-based devices. Typical on-chip dispersion schemes include the use of photonic crystals, holographic elements, transmission gratings, self-focusing transmission gratings, arrayed waveguide gratings, and metasurfaces, and have been discussed in a recent review [127]. These typically suffer from low resolution due to small path lengths, compared to their traditional, bulky counterparts. A popular on-chip alternative to dispersive spectrometers is narrowband filters, where the filter wavelength is very often easily tunable. They can be ultra-compact, due to their planar nature and negligible path length. Both plasmonic and dielectric filter implementations in the near-infrared and mid-infrared have been shown in [133,134]. Of particular interest are ring resonator filters, as already demonstrated in [135]. A typical ring resonator spectrum contains several resonance peaks, separated by the free spectral range, which limits the spectral bandwidth of the filter. One recent demonstration of the integration of a ring resonator with a distributed Bragg reflector has shown the feasibility of isolating a single ring resonance line and making it more robust against thermal drift [136]. For the Raman spectroscopic system, arrayed waveguide gratings and low-loss microrings have been proposed, to function as UV spectrometers [137]. Hartmann et al. demonstrated a waveguide integrated broadband spectrometer, based on random scattering events in disordered medium, whose functionality extends through both the visible and NIR regions [138] and could be used in integration into a Raman spectroscopic system.

Integrated alternatives to Fourier transform spectrometers, based on waveguides, allow not only for system miniaturization but also for the complete elimination of moving parts. The first approach was to introduce multiple Mach-Zender interferometers (MZIs) of different pathlengths to generate a spatially varying interference pattern, also known as spatial heterodyne spectrometers (SHS) [139,140]. These have been demonstrated through the use of an array of tightly coiled spiral waveguides [141]. Other implementations include photonic circuits governed by spatial multiplexing among different interferometers with increasing varied path length, culminating in outputs coupled to a linear detector array [142,143]. A similar detection scheme has been shown using a single MZI and, hence, a single detector, where the electro-optic modulation in lithium niobate waveguides or thermo-optic modulation by using micro heaters was used to tune the optical pathlength of the interferometric arm [144]. Another approach toward miniaturized FTS was to use counter-propagating beams from two waveguides and allow them to interfere to generate a standing interference pattern, also known as stationary wave-integrated FTS, as first demonstrated by Coarer et al. [145]. In this scheme, the spatial interference pattern across the entire length of the waveguide is recorded with the help of many detection elements placed in close proximity to the waveguide. Since the resolution of such a spectrometer depends on the number of the detector elements and the length of the waveguide, difficulty in placing a sufficient number of detector elements results in low resolution. Nie et al. mitigated this issue by generating the interferogram by overlapping the evanescent fields of two co-propagating waveguide modes, thus effectively stretching the interferogram and allowing high resolution with large bandwidth and a low footprint [146].

## 3. Waveguide-Enhanced IR Absorption Spectroscopy

### 3.1. Configurations and Integration

To date, the most common configuration for optical sensing involves free-space coupling between the laser, waveguide, and detector by using bulk optics or microlenses. While conceptually simple, free-spacing coupling suffers from low coupling efficiency due to mode mismatch and is sensitive to vibrations and misalignment, thus affecting the robustness and compactness of the sensor. The usage of spot size converters or couplers has addressed some of the aforementioned problems [147]. At the same time, fiber-based coupling methods have also been used to improve the robustness and stability of the setup. This includes fiber pigtailed lasers or the direct coupling of a free-space beam into a fiber through a fiber collimator. Nonetheless, such techniques still fall short of an idealized compact and integrated version of the sensor that would fit completely on a single photonic chip and be suitable for mass production at a low cost.

First approaches for integration include hybrid integration, heterogeneous integration and monolithic integration. Even in integrated setups, inefficient coupling has been the major problem. This typically stems from the mode mismatch and misalignment between the active and passive sections of the chips, i.e., the laser cavity and the passive waveguide. For improving the coupling efficiency between the waveguide and the light sources, the usage of distributed Bragg reflectors (DBR) or appropriate mode profile engineering has been proposed [148]. Separate prototypes of integrations of the laser and the waveguide, the waveguide and the detector, the laser and detector are found in the literature; however, complete integration of all the three components in a single chip is still an active and ongoing pursuit, with very few actual demonstrations. The following sections describe recent efforts in this area.

#### 3.1.1. On-Chip Light Sources and Passive Waveguide Integration

**Figure 2:**
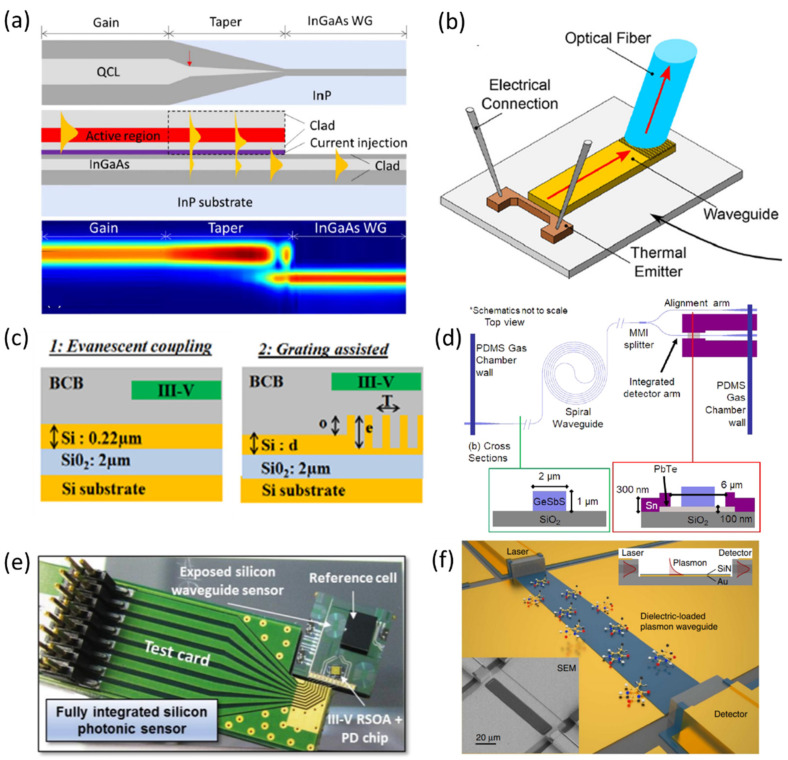
Integration schemes for waveguide-based IR absorption spectroscopy. (**a**) Integrated QCL source with a passive InGaAs waveguide (reproduced from [151] © 2021 Optical Society of America). (**b**) Integration of a waveguide sensor with a MIR thermal emitter (reproduced with permission from [154]). (**c**) Integration of a p-i-n-based GaInAsSb photodetector with a passive waveguide through evanescent and grating-assisted coupling (reproduced from [155] © 2021 Optical Society of America). (**d**) Monolithic integration of a PbTe detector, coupled with a spiral waveguide (reproduced with permission from [156]). (**e**) Prototype of a fully integrated silicon photonic sensor with laser, waveguide, and detector for sensing in the near-infrared (reproduced with permission from [157]). (**f**) Quantum cascade laser and detector, integrated with a dielectric-loaded plasmonic waveguide for sensing in the mid-infrared (copyright © 2021, the author(s) [153]).

Silicon, as a platform for integrated sensors, has been the most frequent choice due to the existing mature process technology and the inherent compatibility with silicon waveguides. However, due to the absence of efficient light sources based on silicon, the heterogeneous integration of light sources, based on III–V materials, has been the preferred choice. This involves attaching the laser chip to a separate chip, with pre-patterned waveguides and other optical components for sensing and manipulation purposes. Heterogeneously integrated devices have been reported both in the NIR and MIR, and this has been discussed in detail in recent reviews [149,150]. Recent works have also focused on InP integration platforms, demonstrated through QCL integration with InGaAs passive waveguides [151] (Figure 2a) and photonic crystal-based laser source integration with silicon waveguides [152]. As a rare example of monolithic integration, QCLs integrated with plasmonic waveguides were demonstrated by Schwarz et al. [153]. In addition, Consani et al. [154] demonstrated the integration of a waveguide sensor with a MIR emitter for CO_2_ sensing, showing that it is possible to abstain from using expensive laser sources and instead use a cheap thermal source (Figure 2b). In their case, the emitted light was broadband, requiring the use of filters on the detector side, but recent advances in narrowband filters and metamaterials emitters can eliminate the use of additional filters.

#### 3.1.2. Passive Waveguide and Detector Integration

Many demonstrations of integrated systems combining a detector and a passive waveguide involve integration of the active detector element, either monolithically or by the hybrid bonding or transfer of the active detector material. In particular, p-i-n photodiodes and 2D materials have been used for the active detector element to achieve compactness. GalnAsSb-based p-i-n photodiodes interfaced with SOI waveguides, either through gratings or evanescent coupling, have been demonstrated at 2.29 μm [155,158] (Figure 2c). Similarly, an InAs_0.91_Sb-based p-i-n photodiode has been shown at 3 μm, integrated on the output grating couplers of a spectrometer [159]. In both cases, heterogeneous integration through adhesive bonding was used. Su et al. fabricated an on-chip waveguide integrated device with monolithically integrated PbTe detector film for the detection of methane at 3.3 μm [156] (Figure 2d). For longer wavelength regions, graphene photodetectors integrated with silicon waveguides have been demonstrated [160]. Yazici et al. showed the integration of a MEMS-based broadband infrared thermopile sensor attached through flip flop bonding with an SOI platform integrated with input and output grating couplers [124]. In all these demonstrations, the active area of overlap of the optical mode with the detector element is still small, resulting in lower sensitivity. Highly sensitive detection needs to increase the detector’s active area, which, unfortunately, also increases the dark current and thus decreases the signal-to-noise ratio. In order to counter this, ridge waveguide-based detection has been used, where the entire length of the detector element acts as the active material for enhanced detection capability and has particularly been used in context with ICDs and QCDs. In order to improve the performance further, distributed Bragg reflectors (DBR), high reflectivity coatings, or simply etched or cleaved facets that ensure multiple passes through the active region have been proposed for a waveguide-integrated ICD. Such a design simultaneously increases quantum efficiency, as well as reducing dark current [148].

#### 3.1.3. Integration of All Three Components

Among very few works describing prototypes of the on-chip integration of all components, Zhang et al. demonstrated a complete integrated setup involving a fully integrated NIR photonic chip sensor, mounted on a PCB test card, with an on-chip laser, dual photodetectors, reference cell, and an evanescent field-based sensing waveguide on a single silicon substrate. With this device, the authors showed methane sensing with a sub-100 ppmv∙Hz^−1/2^ sensitivity [157] (Figure 2e). While this sensitivity still lags behind the state of the art, the fully packaged nature of their demonstration is an important milestone and paves the way for fully integrated devices, particularly in a longer wavelength region. In the MIR, Benedikt Schwarz’s work at TU Vienna and Ray Chen’s group in UT Austin have shown great progress toward fully developed sensors integrated with QCL and QCD. Schwarz et al. [161,162] demonstrated the integration using QCL technology, relying on the bifunctional functionalities of the active region to work as both laser and detector. Coupled with a dielectric loaded plasmonic waveguide, they exhibited a complete system with liquid sensing capability [153] (Figure 2f). Even though a high-power emission was observed, the detector sensitivity was poor in these bifunctional structures. Later demonstrations separated the functions of the laser and detector, allowing their independent design and optimization [163]. In parallel, in order to extend the lasing wavelength to below 6 μm and simultaneously enable low power consumption, high sensitivity, and sufficient design flexibility, subsequent work focused on ICL technology and integrated setups have been demonstrated for 3.1 μm [164]. On the other front, Ray Chen’s group has shown a sensor with QCL and QCD as sources, and detectors integrated with an InGaAs-InP monolithic platform, and gas sensing was demonstrated [165,166] making the pursuit of an on-chip integrated sensor for gas sensing close to reality.

Further advancements have been made through the integration of frequency comb MIR lasers with detectors, which showed ultrafast detection and up to two orders of magnitude lower power consumption, compared to QCLs [167]. In addition, mid-infrared dual-comb spectroscopy is an upcoming area of research, where, through the interference of two mutually coherent mode-locked frequency combs, the absorption spectrum signal can be converted from the optical domain to the radio frequency domain. Dual comb spectroscopy has a fast detection capability with higher resolution and accuracy, making it suitable for gas detection [168,169], and, due to the inherently large bandwidth of the frequency combs, covering even multiple species in parallel [29].

Another strategy to improve the compactness and robustness of the IR spectroscopy setup is to design the sensing functionality within the cavity of the laser, as demonstrated through intra-cavity laser absorption spectroscopy [148,170]. The in situ detection of chemical species within the laser cavity can be monitored directly through the laser’s I-V characteristics, which can even eliminate the use of a separate detector [170].

### 3.2. Applications

#### 3.2.1. Air-Clad

IR absorption spectroscopy on waveguide-based devices has been reported in recent years for various applications in environmental and industrial process monitoring, as well as in the biomedical sector [171,172,173]. In particular, air-clad waveguides have been proven to be useful for IR absorption spectroscopy, to identify and quantify common gases such as carbon dioxide, acetylene, ammonia and methane, some of them with major implications in global warming. However, most demonstrations are still proof-of-concept experiments, testing the capability of the novel integrated sensors. An overview of the air-clad waveguides used for spectroscopy, together with their principal characteristics, is provided in Table 1, while a more detailed description and a discussion of the achieved results are given in the next paragraphs.

**Figure 3:**
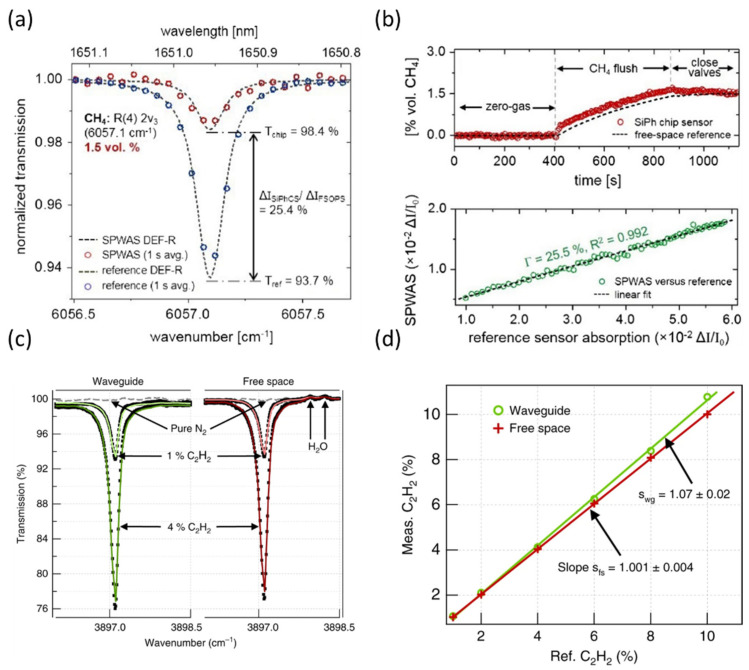
Experimental demonstration of gas detection by WIRAS. (**a**) The spectrum of the methane R(4)2ν_3_ line measured by the waveguide-based integrated spectrometer design by Zhang et al. [157]. The Voight spectral fit for 1.5% methane concentration is shown in red, together with the experimental data. (**b**) Upper plot: experimentally measured methane concentration before and after flushing the chamber with methane. Lower plot: correlation between the absorption measured with the waveguide device and a free-space reference beam, indicating Γ = 25.5% in the waveguide (reproduced with permission from reference [157]). (**c**) Comparison of experimental absorption spectra for 4% and 1% acetylene measured using a free-standing tantala waveguide and a free space beam of identical path length, reproduced from Vlk et al. [69]. (**d**) Correlation of the measured concentration to the reference concentration of data in (**c**). The slope gives Γ = 107% (reproduced with permission under a Creative Commons Attribution 4.0 International License. http://creativecommons.org/licenses/by/4.0/ assessed on 9th September 2021).

The silicon-on-insulator (SOI) platform has been the most popular choice for integrated gas sensor applications in both NIR and MIR. Ranacher et al. [174] demonstrated detection of CO_2_ down to a concentration of 500 ppm with polysilicon strip waveguides on silicon dioxide at 4.26 μm. From the measurements, the confinement factor was estimated to be in the range of Γ = 14–16%, and losses down to 3.98 dB/cm were reported. Silicon strip waveguides were also used for the detection of acetylene and methane by Jin et al. [175]. The group fabricated a 1-cm long waveguide with a thickness of 1 μm, which presents a good compromise between coupling efficiency and evanescence field confinement. The losses were determined to be 1.74 dB/cm and the simulated evanescent field ratio (EFR) was around 13% (EFR does not take into account the group index of the mode; so, although related, this should not be taken as a synonym of the confinement factor). Although the limit of detection was not calculated and the lowest concentration measured was 25% for both gases, the experimental results indicate that a concentration down to 5% could be quantified. The SOI platform was also chosen by Tombez et al. [176], with methane gas as the target analyte. They successfully increased the confinement factor by using TM polarization with a simple strip waveguide to 25.5% and achieved losses near to 2 dB/cm when operating at 1650 μm. The results are shown in Figure 3a,b. Two years later, the group took a breakthrough step toward integration, as discussed in the previous section, being able to successfully integrate a 20 cm spiral waveguide with a 15% confinement factor to a source and a detector on a single chip.

Due to the light absorption in silicon dioxide bottom cladding at wavelengths over 3.5 μm, silicon-on-nitride (SON) and silicon-on-sapphire (SOS) appeared as an alternative to the commonly used SOI [187,188]. SOS has a transparent window of up to 5.5 μm and a high refractive index contrast between the core and the cladding. Chen and collaborators compared experimentally the performance of photonic crystal waveguides (PCW), slot waveguides and strip waveguides on sapphire. Despite the theoretical 1- to 100-fold slow-light driven improvement in the confinement factor, PC waveguides exhibited only slightly higher light–analyte interaction compared to slot waveguides but they were significantly better than strip waveguides. The same group developed PC waveguides even further [183]. Three PCW designs were fabricated in silicon on sapphire: a regular line-defect PCW (so-called W1 waveguide), a holey PCW (HPCW) with smaller diameter holes etched within the light defect, and a slot PCW, wherein a rectangular slot is etched at the center of the PCW. The designs were optimized for 3.43 μm wavelength to quantify xylene and triethyl phosphate (TEP) vapors. In the case of the slot PCW, the authors simulated that the slow light effect, coupled with the high evanescent field confinement in the slot, should reduce the required absorption path length by a factor of 1000 compared to strip or rib waveguides. Although the authors observed a detectable signal change when the waveguide was exposed to 10 ppm TEP with an 800 μm long HPCW, the increase in sensitivity due to the slow light effect is difficult to quantify. The measurements only tracked the total power loss at one wavelength instead of spectrally resolved detection; therefore, the band diagram shift due to the refractive index or temperature variations may affect the signal in the sensitive slow-light regime as well.

As an alternative to CMOS-compatible materials, Charrier et al. [179] reported chalcogenide strip waveguides over silica and calcium fluoride. The strip waveguides presented by the group showed losses as low as 0.4 dB/cm at 1.55 μm but the waveguides were tested in solution and not for gas detection. In a similar work, Han and collaborators [178] fabricated a chalcogenide glass (Ge_23_Sb_7_S_70_) strip waveguide over silica. The 2-cm spiral waveguide showed an air confinement factor of 8%, losses of around 7 dB/cm, and a limit of detection of 2.5% for methane at 3.3 μm [143]. However, the comparably high detection limit is due to a broad-band laser source that cannot resolve the narrow methane lines; a better-suited single-mode continuous-wave laser, such as ICL, would enable much better performance. Finally, Agarwal’s group used the chalcogenide platform to develop a monolithic-integrated on-chip MIR methane sensor [156]. They demonstrated a 5 mm long spiral chalcogenide strip waveguide (Ge_23_Sb_7_S_70_) capable of sensing methane at 10,000 ppm. The design has a similar configuration to the previous report and provides a 1 cm^2^ footprint sensor with both the waveguide and an integrated detector. The losses of the waveguide were measured to be 8 dB/cm, and the air confinement factor, 12.5%, according to simulations.

To further decrease the losses and increase the confinement factor, air-suspended waveguide structures were frequently used over the last few years. Lai et al. [182] proposed photonic crystal slot waveguides for methane detection in the NIR, capable of detecting methane absorption signatures down to several hundred ppm. Dicaire et al. [80] developed a 1.5 mm-long suspended GaInP photonic crystal waveguide and used acetylene to demonstrate its spectroscopic performance. The group indices in the waveguide were 1.5 to 6.7 for the TM and TE modes, while the experimentally obtained confinement factors were 100% and 31%, respectively. The fact that the interaction did not scale with the group index (i.e., the slow-down factor) was due to the considerably larger evanescent field ratio of the TM mode. Based on this result, the authors stress that not only a high group index but also a high evanescent field ratio must be addressed for strong light–analyte interaction, the latter being often neglected in works on photonic crystal waveguides for sensing. The waveguide design also included mode adapters on both end facets to gradually couple into the slow-light mode and thus reduce the Fabry–Perot oscillations. Chen’s group [63] designed and fabricated fully suspended InGaAs waveguide devices with holey photonic crystal waveguides and sub-wavelength grating cladding waveguides for the mid-infrared sensing of ammonia at λ = 6.15 μm (Figure 1b,c). The propagation losses for the two waveguide types were 39.1 and 4.1 dB/cm, the light–analyte overlap was calculated to be 12% (TE) and 10% (TM), lengths, 1 and 3 mm, and the group indices, 39 and 15, respectively. Both waveguides were capable of detecting 5 ppm ammonia; nevertheless, no spectroscopy was performed during the measurement. Changes in power were tracked after flushing ammonia at a constant wavelength, leaving the results susceptible to interference from changes in the environment, including refractive index changes or temperature variations. Ranacher and collaborators [84] designed and fabricated a polysilicon waveguide on a silicon nitride membrane suspended over silica walls. They achieved a 19.5% confinement factor at 4.23 µm. The 1 cm-long waveguide was deployed for CO_2_ detection, with the lowest detected concentration down to 5000 ppm. However, as in the previous work, only the signal drop at one wavelength was recorded and no spectral scan across a CO_2_ absorption line was performed. Gylfason’s group [73] also developed a silicon self-standing waveguide, operational at 4.2 µm wavelength, for CO_2_ gas sensing. Their waveguide was designed as a Si beam, partially suspended 3 µm above the Si handle substrate and supported by tapered SiO_2_ pillars (Figure 1d–f). The waveguide had a large confinement factor of 44%, a total length of 0.5 cm, and losses of 2.9 dB/cm; the CO_2_ detection limit was estimated to be 350 ppm. Although the measurement sampled absorption across a spectral line, unfortunately, the spectroscopy data that was provided had very few points to justify the fitting, considering that fringes might be present. The same group was able to measure CO_2_ on ring resonators by dispersion spectroscopy [181]. As established by the Kramers–Kronig relationship, a strong variation in absorption implies a sharp change in the real part of the refractive index. The group proved that ring resonators based on suspended rib waveguide, with an air confinement factor of 50%, were able to quantify amounts as low as 1000 ppm of carbon dioxide. In 2021, Vlk et al. [69] reported a thin-film suspended tantala rib waveguide for acetylene detection in the MIR. They simulated and proved experimentally that the waveguide is able to achieve a confinement factor of 107%, and thus surpass free-space light–analyte interaction by the combining of a high evanescent field and a group index larger than unity (Figure 1g,h and Figure 3c,d). The authors also proved that the design can suppress the fringes from facet and defect reflections and improve coupling efficiency. The still relatively high losses of 6.8 dB/cm were mainly attributed to absorption into the tantala film.

To take advantage of the field inside the waveguide in addition to the evanescent field, a mesoporous waveguide for IR spectroscopy was reported by Datta and co-workers [180]. Titania rib waveguides over silica bottom cladding, with 52% field confinement in the waveguide core, were fabricated and tested, using acetylene as a calibration gas. They experimentally confirmed that the mesoporous material enables the gas to rapidly diffuse into the core of the waveguide while maintaining a rather low loss of 2 dB/cm. Nevertheless, the presence of O-H groups on the large surface area of the pore network impairs transparency over time. This problem has been partially overcome by annealing and functionalization [177,189].

#### 3.2.2. Cladding

Top cladding on single-mode waveguides for IR spectroscopy has been only used in aqueous solutions to suppress water background and increase the concentration of the analyte close to the waveguide. Although no reports have been made on the gas phase, the use of cladding to preconcentrate organic compounds from aqueous solution also implies the ability to preconcentrate the same analyte from vapors, as has been described for ATR crystals and QCM sensors [190,191]. Therefore, it is of relevance to cover the topic briefly, as cladding on integrated gas sensors can further push the sensitivity of miniature sensors. Here, we stress that LODs in liquid environments are expected to be much lower than in gasses for the same volumetric unit as the density of molecules per volume is several orders of magnitude higher in the condensed phase.

Polymers and porous silica cladding on integrated waveguides have been reported for the detection of pollutants in water solutions. A PDMS cladding on photonic crystal slot waveguides was tested to detect xylene in water down to 100 ppb *v/v* [185]. The group tested the same device with a 2 μm Su-8 coating to measure xylene and trichloroethylene in water, with a detection limit of 1 and 10 ppb (*v/v*), respectively [186]. Polyisobutylene as a sorbent cladding on chalcogenide strip waveguides was proposed but was not experimentally evaluated [192]. According to their calculations, the cladding can decrease the limit of detection by two orders of magnitude, compared to the waveguide without cladding, due to water background suppression and organic analyte diffusion into the cladding. In another work, germanium rib waveguides were coated with a mesoporous silica coating templated with cetyltrimethylammonium bromide (CTAB) and post-grafted with hexamethyldisilazane [177]. This sensor was used to determine the concentration of toluene in water in the 6.5–7.5 µm wavelength range. It is noteworthy to mention that the use of high refractive-index core material (GOS) led to a very low field confinement factor in the cladding of only around 1% when deposited on a strip waveguide. The resulting sensitivity is consequently considerably lower than with other platforms, e.g., silicon nitride rib waveguides are able to confine light in the mesoporous cladding up to 25% [193] and slot waveguides are able to increase this percentage to 36% [76,194].

Both organic polymers and mesoporous inorganic coatings were previously deposited on ATR crystal for the detection of volatile organic compounds (VOC) from vapors [107,195] and no limitation exists to perform the same experiments on single-mode waveguides. However, the possibility of increasing sensitivity for low-weight gases other than VOC by absorption or preconcentration is dependent on the availability of material designs specific to the task. Typically, these materials hold pockets properly matching the targeted molecule size and functional groups. Examples of such materials are molecularly imprinted polymers or composite materials with cage-like organic molecules, such as cryptophanes, able to match the size of methane and halogenated analogs [196]. Alternatively, reactive centers with high selectivity toward specific reactants such as platinum nanoparticles have been described [99]. Although, still, no reports can be found on specific cladding for integrated infrared spectroscopy for the pre-concentration of gases such as carbon dioxide, methane, or acetylene, some examples can be found in works on ATR crystal [197], quartz crystal microbalance [198,199], and refractive index gas sensors [96,99,200].

## 4. Waveguide-Enhanced Raman Spectroscopy

### 4.1. Configuration and Integration

On-chip integration of Raman spectroscopic systems generally requires the enhancement of Raman scattering for greater efficiency, and, at the same time, the scattered light needs to be collected over a small area and a small solid angle (also known as the étendue) to maintain the small size of the device [77]. Single-mode waveguides can provide strong enhancement over a small volume, and therefore constitute an optimal solution for chip-integrated Raman spectroscopic systems. Compared with diffraction-limited systems, waveguide-integrated Raman systems with strong optical field confinement can provide a stronger enhancement of the signal by a few orders of magnitude, allowing for much higher detection sensitivity. Further improvement in signal enhancement can be brought about through the use of nanoplasmonic antennas integrated with waveguides [201].

**Figure 4:**
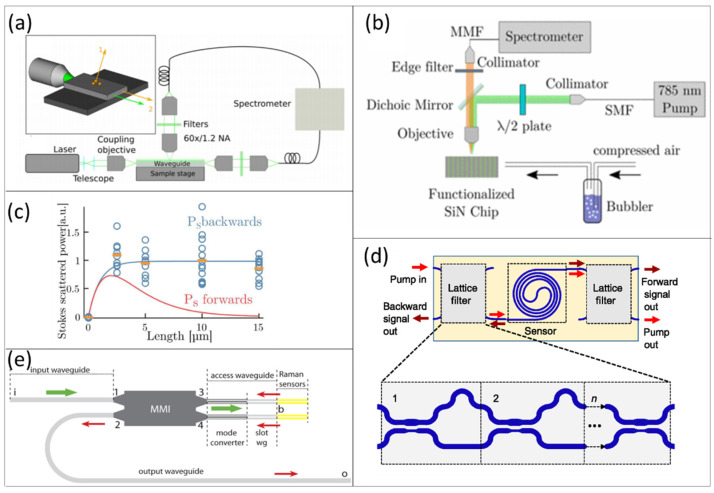
Different configurations as reported for waveguide-enhanced Raman spectroscopy. (**a**) Free space butt coupling of a laser into a waveguide chip for Raman spectroscopy. The figure shows the collection of the scattered signal from both the top surface and from the end facet in a forward configuration (reproduced from [52] licensed under a Creative Commons Attribution 4.0 International License http://creativecommons.org/licenses/by/4.0/ (accessed on 25 October 2021)). (**b**) Backward collection of the Raman scattered signal from a waveguide (reproduced from [203] © 2021, the author(s)). (**c**) Dependence of the scattered signal on the waveguide length for forward- and backward-scattering (reproduced from [48] © 2021, Optical Society of America). (**d**) On-chip scheme to separate the pump beam, the forward- and the backward-scattered signal through the use of lattice filters (reproduced from [204] © the authors). (**e**) WERS setup with a nano-plasmonic waveguide. The design minimizes the background signal, while the use of MMI helps in separating the input pump and the output Raman signal (reproduced from [205] © 2021, Optical Society of America).

For Raman spectroscopy, similarly to IR systems, free-space butt coupling through an objective lens, prism-based coupling, and fiber-mediated coupling have been the prominent mechanisms for introducing the light into the waveguides. On the other hand, collection from waveguides can be performed either from the waveguide top surface or from the waveguide facet, which can be in either a back-scattered or forward-scattered configuration (Figure 4a,b). The collection efficiency from the waveguide facet is generally much higher and has been shown to be about 40 times more efficient than that from the surface [202]. As a consequence, this requires less integration time than that from the waveguide surface [28]. However, signals from the surface can provide additional spatially resolved information [52].

While free-space coupling with a high numerical aperture objective provides good coupling efficiency, it also introduces vibrations and critical alignment steps and is unsuitable for use in compact setups. Fiber-mediated coupling solves some of these issues, but it introduces a spurious background signal, including fluorescence and Raman scattering generated from both the input and the output fibers. Kita et al. aimed to eliminate this effect by collecting only the backscattered light. This tactic removes much of the forward-propagating pump beam, which results in a higher signal-to-noise ratio [47]. The collection of the backscattered beam also has the advantage of having virtually no waveguide length limit due to the propagation loss of the waveguide, even though the contribution to the scattered signal for waveguides of lengths longer than $2/\alpha_{p}$ is negligible, where $\alpha_{p}$ is the propagation loss of the waveguide. In return, the forward-scattered light collection efficiency generally has a maximum for a particular waveguide length, beyond which the propagation loss dominates, thus reducing the collected signal power (Figure 4c). A different approach to eliminating the influence of the background has been to integrate edge couplers and waveguide filters onto the chips, as demonstrated by Tyndall et al. [204,206] (Figure 4d). An array of polarization maintaining single-mode fibers is aligned directly to the waveguide facets through edge couplers; the subsequent use of lattice filters helped in separating the background and collecting both the forward- and backward-propagated Raman scattered light at separate outputs. Other on-chip elements, such as a grating-assisted contra-directional coupler, have also been proposed to reject the pump beam by directing it to a separate bus waveguide, resulting in a very high extinction ratio [207]. Nonetheless, the use of dielectric waveguides still introduces some photon background, likely arising from localized thermal fluctuations, which has been difficult to get rid of. The use of a nano-plasmonic slot waveguide, combined with a multi-mode interferometer (MMI) and backward Raman collection, has been shown to mitigate this problem [205] (Figure 4e).

Spontaneous Raman scattering signal intensity grows linearly with the average power of a continuous-wave pump laser; this has been a major bottleneck in improving the Raman signal, particularly in a miniaturized system where it is not possible to increase the pump power indefinitely without causing substantial damage. On the other hand, coherent Raman scattering (CRS) is a third-order non-linear phenomenon involving two laser beams, the pump, and the Stokes. When the difference in frequency between both respective lasers equals that of a specific vibrational (or rotational) transition, the probability of this transition is resonantly enhanced. CRS is capable of improving signal by many orders of magnitude and is typically implemented in two configurations, coherent anti-stokes Raman scattering (CARS), and stimulated Raman scattering (SRS). Of these two techniques, SRS has been shown to be more promising, particularly in the context of waveguide-based Raman sensors. The reasons behind this are a simpler phase-matching relationship between the two lasers, a linear dependence of the Raman spectra on concentration, and an enhancement of the signal due to self-heterodyned detection. However, in spite of these advantages, increased shot noise severely limits the performance, only providing a modest increase of the Raman signal, as demonstrated by Zhao et al. [208]. On the other hand, other techniques such as cavity-enhanced Raman spectroscopy (CERS) and Purcell-enhanced Raman spectroscopy (PERS) have been recently used to resonantly enhance the laser beam power, as well as to improve the rate of Raman scattering, resulting in having higher laser beam–analyte interaction lengths [209,210,211]. These allow for sufficiently low pump powers and, in combination with their small size, may play a very important role in constructing miniaturized devices in the future.

Despite the above-reviewed efforts, the complete photonic integration of the Raman spectroscopic system is still in its infancy, primarily due to the fact that WERS requires high-power monochromatic light sources at visible or near-infrared wavelengths, high-extinction ratio filters, and sensitive detectors. Beyond this, the suppression of unwanted fluorescence and background from the waveguide material needs to be improved to match the performance of bulk Raman systems [212].

### 4.2. Applications

#### 4.2.1. Air-Clad

The first steps toward the use of waveguides for Raman spectroscopy came more than 40 years ago, with the use of planar waveguides to characterize polymeric thin films [213]. It was observed that the enhancement of Raman excitation in thin films resulted from maintaining a high excitation intensity over an increased scattering volume of the analyte [214]. Further studies revealed that high-index waveguides yielded the greatest field enhancement for Raman excitation at the surface of an optical waveguide [215]. To better quantify the waveguide performance for Raman sensing, Baets et al. defined the so-called conversion efficiency, a parameter that is dependent on both the waveguide geometry and the material. The group corroborated their finding experimentally with isobutanol at 785 nm [71].

Waveguide-based Raman spectroscopy demonstrations published to date rely on high refractive-index waveguide materials that are transparent in the visible–NIR range, such as silicon nitride and tantalum pentoxide. Similar to IR spectroscopy, the strip, rib and slot waveguides are the most popular designs [65,71,74,216]. Tantala rib waveguides were tested on isopropanol, methanol, and finally, with hemoglobin solutions at physiological concentrations as the first step toward future nanoscopy applications [52]. Silicon nitride strip waveguides were designed in a spiral pattern for WERS at 785 nm and were used to track isopropanol by Baets’ group [71]. Soon after, the same group proposed a design based on slot waveguides, which allows for better light–analyte interaction in the slot and, due to lower optical field confinement in the waveguide material, it also mitigates the waveguide Raman background [212]. The introduction of slot waveguides resulted in a 6-fold improvement in performance for silicon nitride waveguides, compared with strip waveguides [51]. Furthermore, Baets and collaborators studied theoretically the influence of the refractive index and polarization on Raman conversion efficiency, concluding that the TE polarization in slot waveguides with a high refractive index contrast presents the highest value from the three designs discussed [217]. Despite the success of Si_3_N_4_ spiral waveguides, the Raman background and fluorescence signal in the material still pose a serious limitation to detection sensitivity. Therefore, the low density of molecules, such as those found in the gas phase, has not been detected in non-functionalized (or unclad) waveguides so far, and most reports have been on liquid samples.

The use of plasmonics has also been proposed and widely studied, in order to increase the Raman scattering yield. Interaction between nanoplasmonics antennas and waveguides was described both theoretically and experimentally by several authors [218,219,220] and the implications for sensing have been explored. Although great advances have been made in the field, including integrated plasmonic moieties to the waveguide [48,50], the performance of such devices in comparison to traditional WERS has not shown clear advantages. Plasmonics brings signal enhancement, but it simultaneously increases losses, thus limiting the propagation length to several micrometers in comparison to the centimeter-length scale achieved by common WERS. Therefore, the Raman conversion efficiency results in comparable values, being slightly superior for a 4-cm slot waveguide than 15-μm hybrid plasmonic waveguides. The main advantage of hybrid waveguides has been the reduced spurious Raman background generated from the core of the dielectric waveguide. Nevertheless, neither of these approaches proved sensitive enough for gas sensing.

Stimulated Raman scattering (SRS), as a strategy to increase the Raman signal, was tested experimentally on silicon nitride waveguides by Baets et al. [208]. The signal was enhanced but so was the noise, with a resulting signal-to-noise ratio only slightly improved compared to spontaneous Raman scattering. This mediocre result has been attributed to sub-optimized design and equipment, while more significant improvement with a better-optimized system was not excluded.

#### 4.2.2. Clad/Functionalized

**Figure 5:**
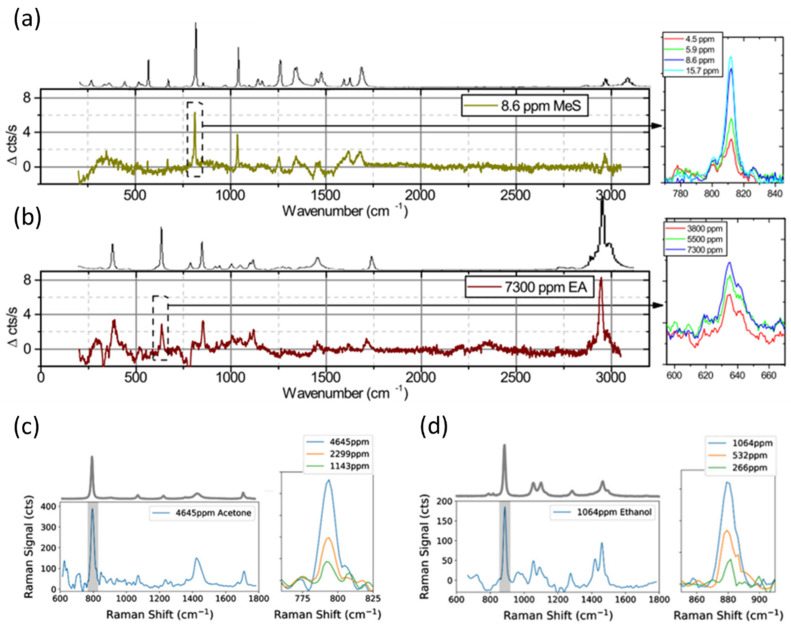
Examples of WERS spectra. Experimental Raman spectrum after background subtraction for vapors absorbed in the cladding (HCSFA2 (**a**,**b**), Ms-HMDS (**c**,**d**)). In each plot, the reference spectrum is placed above the experimental one, and the selected peak used to track different concentrations is shown to the right. Reproduced with permission from reference [193], © 2021, Optical Society of America, and reference [194], © 2021, Optical Society of America.

The use of polymer cladding, with the ability to preconcentrate gases and, thus, increase the Raman signal, has been shown recently in several reports. Holmstrom et al. [193] covered Si_3_N_4_ rib waveguides with a hyperbranched carbosilane fluoroalcohol-based sorbent polymer (HCFSA2). These sensing properties were first tested with ethyl acetate (EA), methyl salicylate (MeS), and dimethyl sulfoxide (DMSO), listed in increasing order of their partitioning coefficient into HCSFA2 (Figure 5a,b). These analytes, exhibiting carboxylate and sulfoxide structures, work as substitutes for the more toxic phosphonate esters, which are well-known warfare agents with a major effect on the nervous system. Later on, the detection of dimethyl methyl phosphonate (DMMP), diethyl methane phosphate (DEMP), trimethyl phosphate (TMP), and triethyl phosphate (TEP) in HCSFA2 over a single-mode waveguide was also studied by Tyndall and collaborators [221]. They reported a limit of detection of approximately 5, 10, 50 and 50 ppb, respectively. The limit of detection was related to the increase in the basicity of the compounds and the affinity to the acid–polymer cladding. The same group recently proposed alternative coatings for the waveguide-enhanced Raman spectroscopy of trace chemical warfare agent simulants. DMMP was chosen to compare the performance of three alternative coatings to HCSFA2: carbosilane chain polymer poly(methyl 2-butanol, 1,1,1-trifluoro-2-(trifluoromethyl)) siloxane (PMBTTS); 2,2-bis(4-hydroxy-3-propyl phenyl) hexafluoropropane (o1pBPAF); and fluoro-polyol (FPOL). Each sorbent is a hydrogen-bond acid designed to target hydrogen-bond basic vapors. The group proved for this particular analyte that o1pBPAF has a better performance over HCSFA2 [222].

Alternatively to polymer cladding, sol-gel-based materials were tested for WERS by Haolan Zhao et al. [194]. Motivated by the two orders of magnitude of improvement for the determination of benzonitrile, valeronitrile and cyclohexanone from water, they tested the performance of a mesoporous silica cladding post-grafted with hexamethyldisilazane against solvent vapors (VOCs) in a gaseous matrix. The resultant hydrophobic coating could absorb VOCs, particularly ethanol, acetone and isopropyl alcohol (Figure 5c,d). This cladding was used to coat a single- and a double-slot silicon nitride waveguide. The confinement factor was estimated to be 36% and 32%, respectively, at a 785 nm wavelength. The single-slot waveguide LOD for isopropanol, ethanol and acetone was 53, 157 and 594 ppm, respectively [194]. The double-slot waveguide device could determine the concentration of isopropanol vapors down to 808 ppm [203]. Table 2 summarizes the most relevant information from clad WERS gas sensors discussed so far.

Despite the undeniable increase in sensitivity enabled by the use of enrichment cladding, several drawbacks can be identified for both polymers and sol-gel layers. VOCs at standard pressure and temperature are liquids according to the phase diagram, readily susceptible to condensation in the cladding, as can be seen in the broad absorption bands analyzed in most of these reports [223]. The broad spectral features are susceptible to cross-sensitivity and limit the sensor selectivity, i.e., the capability to discriminate different compounds in complex matrices [194]. In addition, signal enhancement comparable to VOC cannot be easily achieved for gases with critical temperatures below or near ambient temperature, such as methane, carbon dioxide, or acetylene.

## 5. Summary of Current Technology–Comparison with Refractive Index Sensing

**Figure 6:**
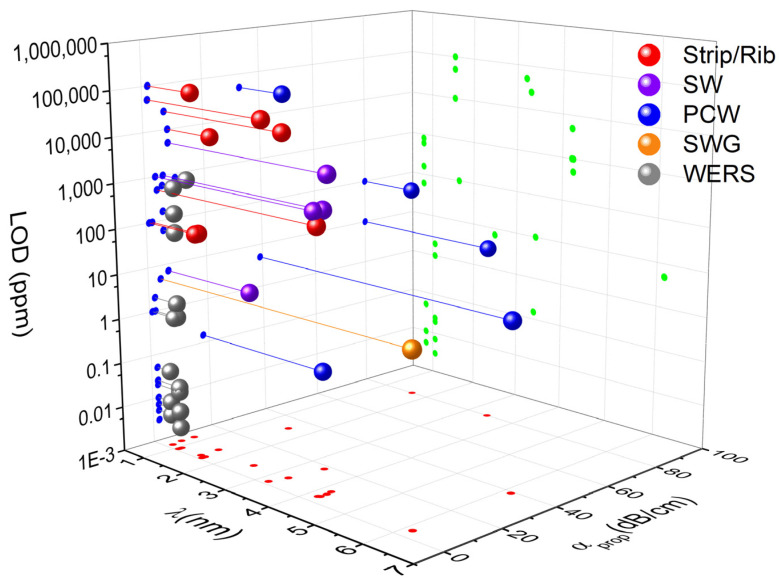
Technology map. A 3D plot of various representative sensing structures, categorized according to their working wavelength l (x-axis), propagation losses α_prop_ (y-axis), and LOD (z-axis). Legend: strip/rib waveguides (strip/rib), suspended waveguides (SW), photonic crystal waveguides (PCW), subwavelength gratings (SWG), clad waveguides used for WERS (WERS).

We summarize the reviewed literature with a technology map (Figure 6). The technology map is a 3D plot, where each data point represents one particular sensing structure, plotted against its LOD, operation wavelength l, and propagation loss, a_prop_. Different sensing techniques are color-coded to show the general trends for each sensor family. Absorption spectroscopy sensors based on strip or rib waveguides (red) are located in the upper left corner, with LODs above 100 ppm. Lower LODs have been achieved with suspended waveguides (purple) at longer wavelengths, with the most sensitive among them capable of detecting a gas concentration of 7 ppm. Lower LODs are owing to clad systems. Raman measurements (grey) of VOC (liquid at normal standard conditions) present limits of detection two orders of magnitude lower, due to the enrichment properties of the cladding. These devices operate at a shorter wavelength region, closest to the left corner. Subwavelength gratings and photonic crystal-based devices (orange and blue, respectively) were able to detect gases such as NH_3_ with a LOD down to 0.150 ppm and are located at the back of the technology map due to high losses (non-reported propagation losses were estimated based on the length of the used waveguide).

## 6. Outlook and Future Perspectives

With this work, we strove to provide a comprehensive review of on-chip waveguide-based IR-absorption and Raman spectroscopy sensors for gas sensing. We discussed the main components, including new and sophisticated waveguide designs, as well as the latest advances in the domain of spectroscopic sensor integration.

From the IR absorption-sensing perspective, traditional simple rib and strip waveguides have evolved in design and processing into self-standing designs, with a corresponding increase in confinement factor from one digit to more than 100%. This dramatic enhancement in confinement factors, together with transition to MIR wavelengths and compatible nanophotonic components, brought the limit of detection down by several orders of magnitude since the first waveguide-based sensor reports. In return, integrated sensors capable of detecting small gas molecules below 10 ppm were reported (see Table 1). To reduce the limit of detection even further, slotted photonic crystals, capable of reducing the speed of light while maintaining a high air confinement factor, were proposed and fabricated. Nevertheless, the performance is still limited by the high propagation losses of the waveguides, allowing for only centimeter- or, in the case of photonic crystals, millimeter-long pathlengths.

On another front, the use of enrichment cladding that is compatible with integrated waveguide platforms showed promise for enhancing the sensitivity of WIRAS by the selective absorption and up-concentration of volatile analytes. So far, cladding (applied on WIRAS) has been only used to sample solvents in aqueous environments; however, no restrictions exist to use them with gas matrices as long as the cladding remains transparent within the wavelength range of interest. Further development will imply the use of recognition sites in the cladding to boost the specificity and allow for the enrichment of other gases than VOCs. Enrichment remains a challenging topic for small molecules and, particularly, for gases with critical temperatures below room temperature, such as the majority of greenhouse gases including methane and CO_2_. Some progress has been made in this direction by developing composite cladding, with cage-like molecules as trapping sites, but the specificity, the transparency, and the processing still need to be matched.

Waveguide-enhanced Raman spectroscopy greatly profits from the high intensity of tightly confined guided modes and from Raman signal collection along the entire waveguide length, increasing both the signal-to-noise ratio and the sensitivity. Nevertheless, the limit of detection of air-clad sensors remains around 50,000 ppm in solution and could not be applied to gases. In a quest to improve this figure, plasmonic structures coupled to waveguides for the surface enhancement of Raman scattering (SERS) were tested, but no great improvement in the performance was reported other than partial background suppression. Only the use of enrichment cladding significantly pushed down the limit of detection. Depending on the Raman cross-section values, the analyte and the cladding, the LOD in a solution can drop down to parts-per-billion.

Comparing WIRAS and WERS, the weak Raman cross-section has limited the WERS sensor performance in comparison with absorption spectroscopy. The absence of direct gas sensing with air-clad waveguides in a Raman configuration and orders of magnitude lower detection limits for absorption spectroscopy, even with the use of cladding, are a direct consequence. The performance of waveguides for both techniques is still limited by losses, while Raman sensors additionally require the careful selection of materials to suppress the spurious Raman background. WERS sensors, however, have a certain advantage over WIRAS for the detection of larger gas molecules, molecules in cladding, or in liquid matrices. Namely, WERS offers the spectral coverage needed to capture the broad spectral features of such analytes and maintain the sensor specificity. Another advantage of WERS is its lower sensitivity to water interference, as Raman spectra typically overlap with a water window spanning from 600 to 2600 cm^–1^, with a minor band at 1600 cm^–1^ [225]. Moreover, WERS systems operate in VIS-NIR, where the photonic materials and fabrication processes are mature, and traditional cladding materials such as polymers or mesoporous oxide films are transparent. In contrast, in WIRAS, strong MIR water absorption bands must be avoided, e.g., by measurement at low pressure and between water absorption lines, or water needs to be excluded by sample preconditioning or hydrophobic functionalization. WIRAS waveguides are also subject to spurious absorption due to residual OH, NH groups, or organic cladding, which is currently the major factor limiting their performance. Finally, both WIRAS and WERS can handle small sample volumes in combination with microfluidics, which is a major advantage of both techniques compared to the bulk systems.

At present, the miniaturization of WIRAS gas sensors is a large step forward ahead of WERS, with the first systems integrating both laser, waveguide, and detector being successfully demonstrated in both the NIR and MIR. The testing of such sensors in practical applications, followed by commercialization efforts, is underway, and first reports on the deployment of integrated absorption spectroscopic sensors within new platforms such as networks or UAVs will likely emerge within a few years. WERS sensors are, on the other hand, more suitable for large-molecule detection and operation with enrichment cladding. We expect an increasing number of works exploring chemical-spectroscopic detection using such sensors, eventually translating into commercial applications in chemical, biological, and biomedical research.

## Figures and Tables

**Table 1:** Overview of works using WIRAS for gas sensing.

Strucuture	Cladding	Analyte-LOD	Γ/EFR	Losses -λ	Advantages -Disadvantages	Ref.
**Strip Waveguides**						
Polysilicon strip waveguides over SiO_2_/Si_3_N_4_	Air	CO_2_ 500 ppm	14–16% TE (EFR)	3.98–5.6 dB/cm 4.23 μm	Simple with moderate confinement factor. Single wavelength measurement.	[174]
Silicon strip waveguide	Air	CH_4_-C_2_H_2_ <50,000 ppm	13% TE (EFR)	1.74 dB/cm 3–3.3 μm	Simple design and fabrication. Low losses. Moderate confinement factor. High LOD. Wavelength Scanning measurement.	[175]
Silicon strip waveguide	Air	CH_4_ < 100 ppm	15% TM (Γ)	Not reported 1.65 μm	Fully integrated chip with a 20-cm-long silicon waveguide. wavelength-scanning measurement.	[157]
Silicon strip waveguides on silica	Air	CH_4_ 100 ppm	25.5% TM (Γ)	2 dB/cm 1.65 μm	High confinement factor for a strip waveguide. Relatively low losses. Low LOD. Wavelength scanning measurement.	[176]
Germanium on silicon strip waveguides	MS- HDMS-water	Toluene 7 ppm	1% (EFR)	2.5–5 dB/cm 6.5–7.5 μm	100–1000× preconcentration. High LOD. Wavelength Scanning measurement.	[177]
Chalcogenide strip spiral waveguide	Air	CH_4_ 25000 ppm	8% (EFR)	7 dB/cm 3.28–3.34 μm	Low confinement factor and high losses. Not CMOS compatible. Wavelength scanning measurement.	[178]
Chalcogenide strip waveguide on silica and CaF_2_	Water	Phenylethyl amine 1800 ppm (mol/mol) (0.1 mol/L) (12 g/L)	5–15% (EFR)	0.4–1 dB/cm 1.52–1.56 μm	Low losses. Not CMOS compatible, low confinement factor. Single wavelength measurement.	[179]
Chalcogenide strip spiral waveguide	Air	CH_4_ 10,000 ppm	12.5% (Γ)	8 dB/cm 3.31 μm	Waveguide and detector integrated on the same chip. Single wavelength measurement.	[156]
TiO_2_ rib porous waveguide on SiO_2_	Air	C_2_H_2_ <100,000 ppm	26% TE (Γ)	2.2–8.5 dB/cm 1.5–1.6 μm	Simple and inexpensive. Highest confinement factor for rib waveguide. Strong fringes. High LOD. Wavelength scanning measurements	[180]
**Suspended Waveguides**						
Polysilicon-on- Si_3_N_4_ membrane over Si/SiO_2_ walls	Air	CO_2_ 5000 ppm	19.5% (Γ)	Not reported 4.23 μm	Complicated fabrication, moderate improvement in confinement factor. Single wavelength measurement. 1 cm long.	[84]
Silicon beam on pillars	Air	CO_2_ < 1000 ppm	44% (Γ)	3–4 dB/cm 4.24 μm	Sophisticated fabrication and moderate losses. High confinement factor. Few wavelength measurements.	[73]
Suspended tantala rib waveguide	Air	C_2_H_2_ 7 ppm	107% TM (Γ)	6.8 dB/cm 2.55 μm	Highest reported confinement factor. Low fringes. Moderate losses. Low LOD. Wavelength scanning measurements.	[69]
Suspended ring resonator	Air	CO_2_ 1000 ppm	50% TE (Γ)	Not reported 4.23 μm	High confinement factor. Original but complicated measurement based on dispersion spectroscopy. Wavelength scanning measurements. Ring length935 μm.	[181]
**Photonic Crystals**						
Photonic crystal	Air	CH_4_ > 100 ppm	Not reported n_g_ = 30	Not reported 1660–1670 nm	High losses restrict the length to 300 μm. Wavelength scanning measurements.	[182]
Photonic crystal slot waveguide	Air	TEP 10 ppm	Not reported	Not reported 3.43 μm	No spectroscopic measurements were made. Changes in temperature and refractive index could not be ruled out. 800 μm long.	[183]
SOI holey photonic crystal waveguide	Air	Ethanol 150 ppb	17% (ERF) n_g_ = 73	Not reported 3.4 μm	9 mm long photonic crystal. Due to single wavelength measurement, the results are susceptible to environmental changes.	[184]
Photonic crystal slot waveguide	PDMS-water	Xylene 100 ppb (*v/v*) (86 μg/L)	Not reported n_g_ = 20	Not reported 1.69 μm	Low LOD, small differences between fabrication and design have significant effects. 300 μm long. Spectroscopic measurement.	[185]
SOI Photonic crystal waveguide	SU8-water	Xylene 1 ppb Trichloroethane 10 ppb (*v/v*)	Not reported n_g_= 23–33	Not reported 1.640–1.680 μm	300μm long. Low LOD. Sigle wavelength measurement for each analyte. The whole device includes a Y-junction combiner, PCW, and MMI.	[186]
Self-standing GaINP Photonic crystal	Air	C_2_H_2_ < 50,000 ppm	100% TM 31% TE (Γ) n_g_ = 1.5–6.7	Not reported 1520–1570 μm	High confinement factor. 1.5 mm long photonic crystal waveguide. High LOD. Wavelength scanning measurements.	[80]
InGaAs self-standing holey photonic crystal	Air	NH_3_ 5 ppm	12% (EFR) n_g_ = 39.3	39.1 dB/cm 6.15 μm	1 mm long. No spectroscopy measurement was presented. The results are susceptible to environmental changes.	[63]
**Subwavelength Grating**						
Subwavelengh grating waveguides	Air	NH_3_ 5 ppm	10% (EFR)	6.15 μm n_g_ = 14.8 4.1 dB/cm	3 mm long. No spectroscopy results were presented. The results are susceptible to environmental changes.	[63]

**Table 2:** Overview of works using clad waveguides for WERS.

Structure	Cladding	Losses (*α_p_*)/λ	Analyte	Γ/ Polarization	Length	LOD	Ref.
Si_3_N_4_ rib waveguide	HCSFA2 FPOL PMBTTS O1pBPAF	1–5 dB/cm 1060–1300 nm	DMMP	Not reported 1064 TM	Spiral length not specified *	3–1000 ppb	[222]
Si_3_N_4_ rib waveguide	HCSFA2	1–2.5 dB/cm 1060–1200 nm	DMMP DEMP TMP TEP	Not reported 832 nm TM	9.6 mm	5, 10, 50, 50 ppb	[221]
Si_3_N_4_ rib wavegudies	HCSFA2	2 dB/cm 980–1600 nm	EA MeS DMSO	25% TE 1064 nm	9.6 mm	1.8 ppm 1 ppm 24 ppb	[193]
Si_3_N_4_ double slot waveguide	MS-HMDS	Not reported	isopropanol	32% TE 785 nm	10 mm	808 ppm	[203]
Si_3_N_4_ slot waveguide	MS-HMDS	5.6 dB/cm	isopropanol, acetone, ethanol	37% TE 785 nm	8 mm	60, 600, 160 ppm	[194]

* The group reported 3.1–5.7-cm long spirals in another publication [224].
